# 1-(2,2-Dimeth­oxy­eth­yl)-8-nitro-1,2,3,5,6,7-hexa­hydro­imidazo[1,2-*a*]pyridin-5-ol

**DOI:** 10.1107/S1600536810033660

**Published:** 2010-08-28

**Authors:** Zhongzhen Tian, Gaolei Wang, Haijun Dong

**Affiliations:** a Shandong Provincial Key Laboratory of Fluorine Chemistry and Chemical Materials, School of Chemistry and Chemical Engineering, University of Jinan, People’s Republic of China; b School of Sciences, University of Jinan, People’s Republic of China

## Abstract

In the title compound, C_11_H_19_N_3_O_5_, the six-membered ring displays a half-chair conformation and the imidazolidine ring is essentially planar (r.m.s. deviation = 0.088 Å). An inter­molecular hydrogen bond between the hy­droxy O atom and a nitro O atom stabilizes the crystal packing.

## Related literature

For related structures, see: Tian *et al.* (2010 ▶); Li *et al.* (2010 ▶). For background to neonicotinoid insecticides, see: Mori *et al.* (2001 ▶); Ohno *et al.* (2009 ▶); Jeschke *et al.* (2008 ▶); Tian *et al.* (2007 ▶).
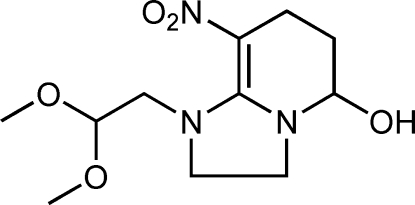


## Experimental

### 

#### Crystal data

C_11_H_19_N_3_O_5_
*M* *_r_* = 273.29
Monoclinic, 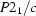

*a* = 11.2337 (6) Å
*b* = 9.0903 (3) Å
*c* = 14.2618 (7) Å
β = 113.124 (6)°
*V* = 1339.36 (11) Å^3^
*Z* = 4
Mo *K*α radiation
μ = 0.11 mm^−1^
*T* = 293 K
0.46 × 0.20 × 0.16 mm

#### Data collection

Bruker APEXII CCD area-detector diffractometer
Absorption correction: multi-scan (*SADABS*; Bruker, 2005 ▶) *T*_min_ = 0.929, *T*_max_ = 1.0
37886 measured reflections
2723 independent reflections
2100 reflections with *I* > 2σ(*I*)
*R*_int_ = 0.033

#### Refinement

*R*[*F* ^2^ > 2σ(*F* ^2^)] = 0.042
*wR*(*F* ^2^) = 0.120
*S* = 1.06
2723 reflections
175 parameters
H-atom parameters constrained
Δρ_max_ = 0.18 e Å^−3^
Δρ_min_ = −0.19 e Å^−3^

### 

Data collection: *APEX2* (Bruker, 2005 ▶); cell refinement: *SAINT* (Bruker, 2005 ▶); data reduction: *SAINT*; program(s) used to solve structure: *SIR97* (Altomare *et al.*, 1999 ▶); program(s) used to refine structure: *SHELXL97* (Sheldrick, 2008 ▶); molecular graphics: *XP* (Sheldrick, 2008 ▶); software used to prepare material for publication: *WinGX* (Farrugia, 1999 ▶).

## Supplementary Material

Crystal structure: contains datablocks I, global. DOI: 10.1107/S1600536810033660/bt5332sup1.cif

Structure factors: contains datablocks I. DOI: 10.1107/S1600536810033660/bt5332Isup2.hkl

Additional supplementary materials:  crystallographic information; 3D view; checkCIF report

## Figures and Tables

**Table 1:** Hydrogen-bond geometry (Å, °)

*D*—H⋯*A*	*D*—H	H⋯*A*	*D*⋯*A*	*D*—H⋯*A*
O1—H1⋯O5^i^	0.82	1.89	2.6966 (16)	169

Symmetry code: (i) 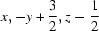.

**Fig. 1.**
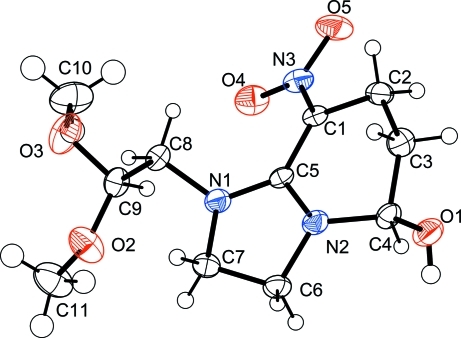
The molecular structure of the title compound with atom numbering scheme. Displacement ellipsoids are drawn at the 30% probability level. The H atoms are shown as spheres of arbitrary size.

**Fig. 2.**
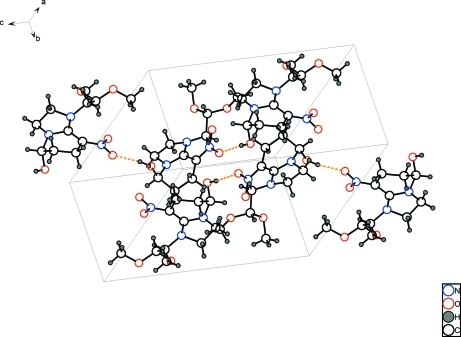
Intermolecular hydrogen bonding in the crystal structure.

## supplementary crystallographic information

### Comment

Neonicotinoid insecticides have rapidly grown and become a new chemical class of insecticides in recent years because of their novel structure and mode of action compared with conventional insecticides (Ohno *et al.*, 2009; Jeschke *et al.*, 2008). We have synthesized a series of new compounds by introducing a tetrahydropyridine ring into the lead structure to improve photostability, in which the title compound exhibited moderate insecticidal activities against pea aphids.

The structure of the title compound is shown in Fig. 1 with the atom-numbering scheme. The title compound is a homologue of (*E*)-1-(2,2-dimethoxyethyl)-2-(nitromethylene)imidazolidine (Li *et al.*, 2010). The six-membered ring displays a  half-chair conformation and the imidazolidine ring is essentially planar (r.m.s. deviation = 0.088 Å). An intermolecular hydrogen bond between the hydroxyl group O atom and the nitro-group O stabilizes the crystal packing.

### Experimental

To a mixture of 1-((1,3-dithiolan-2-yl)methyl)-2-(nitromethylene)imidazolidine (2 mmol) were added olefin aldehyde (2.2 mmol), acetonitrile (20 ml), and a drop of concentrated hydrochloric acid. The reaction was carried out at 40°, and the progress of the reaction was monitored by TLC. After completion of the reaction, the solvent was removed under reduced pressure, and the crude oil was purified by flash chromatography to give the desired product. Single crystals suitable for X-ray analysis were obtained by slow evaporation of a solution of dichloromethane and ethyl acetate of the title compound.

### Refinement

All H atoms were placed in their calculated positions and then refined using riding model with C—H = 0.96–0.98 Å, *U*_iso_(H) = 1.2 (1.5 for methyl groups) times *U*_eq_(C).

### Crystal data

**Table:**

C_11_H_19_N_3_O_5_	*F*(000) = 584
*M**_r_* = 273.29	*D*_x_ = 1.355 Mg m^−^^3^
Monoclinic, *P*2_1_/*c*	Mo *K*α radiation, λ = 0.7107 Å
Hall symbol: -P 2ybc	Cell parameters from 15115 reflections
*a* = 11.2337 (6) Å	θ = 3.0–28.9°
*b* = 9.0903 (3) Å	µ = 0.11 mm^−^^1^
*c* = 14.2618 (7) Å	*T* = 293 K
β = 113.124 (6)°	Prism, colourless
*V* = 1339.36 (11) Å^3^	0.46 × 0.20 × 0.16 mm
*Z* = 4	

### Data collection

**Table:**

Bruker APEXII CCD area-detector diffractometer	2723 independent reflections
Radiation source: fine-focus sealed tube	2100 reflections with *I* > 2σ(*I*)
graphite	*R*_int_ = 0.033
Detector resolution: 16.0355 pixels mm^-1^	θ_max_ = 26.4°, θ_min_ = 3.0°
ω scans	*h* = −14→14
Absorption correction: multi-scan (*SADABS*; Bruker, 2005)	*k* = −11→11
*T*_min_ = 0.929, *T*_max_ = 1.0	*l* = −17→17
37886 measured reflections	

### Refinement

**Table:**

Refinement on *F*^2^	Primary atom site location: structure-invariant direct methods
Least-squares matrix: full	Secondary atom site location: difference Fourier map
*R*[*F*^2^ > 2σ(*F*^2^)] = 0.042	Hydrogen site location: inferred from neighbouring sites
*wR*(*F*^2^) = 0.120	H-atom parameters constrained
*S* = 1.06	*w* = 1/[σ^2^(*F*_o_^2^) + (0.0615*P*)^2^ + 0.2341*P*] where *P* = (*F*_o_^2^ + 2*F*_c_^2^)/3
2723 reflections	(Δ/σ)_max_ = 0.005
175 parameters	Δρ_max_ = 0.18 e Å^−^^3^
0 restraints	Δρ_min_ = −0.19 e Å^−^^3^

### Special details

**Table:**

Geometry. All e.s.d.'s (except the e.s.d. in the dihedral angle between two l.s. planes) are estimated using the full covariance matrix. The cell e.s.d.'s are taken into account individually in the estimation of e.s.d.'s in distances, angles and torsion angles correlations between e.s.d.'s in cell parameters are only used when they are defined by crystal symmetry. An approximate (isotropic) treatment of cell e.s.d.'s is used for estimating e.s.d.'s involving l.s. planes.
Refinement. Refinement of *F*^2^ against ALL reflections. The weighted *R*-factor *wR* and goodness of fit *S* are based on *F*^2^, conventional *R*-factors *R* are based on *F*, with *F* set to zero for negative *F*^2^. The threshold expression of *F*^2^ > σ(*F*^2^) is used only for calculating *R*-factors(gt) *etc*. and is not relevant to the choice of reflections for refinement. *R*-factors based on *F*^2^ are statistically about twice as large as those based on *F*, and *R*- factors based on ALL data will be even larger.

### Fractional atomic coordinates and isotropic or equivalent isotropic displacement parameters (Å^2^)

**Table:**

	*x*	*y*	*z*	*U*_iso_*/*U*_eq_	
N1	0.29875 (12)	0.55756 (14)	0.22689 (9)	0.0425 (3)	
O1	0.63771 (13)	0.70130 (14)	0.20749 (9)	0.0628 (4)	
H1	0.6117	0.7331	0.1491	0.094*	
N2	0.47152 (13)	0.53178 (15)	0.19064 (9)	0.0445 (3)	
C5	0.42919 (14)	0.56183 (15)	0.26436 (10)	0.0371 (3)	
O4	0.38448 (13)	0.73086 (14)	0.41241 (9)	0.0600 (3)	
N3	0.49381 (14)	0.67236 (15)	0.43224 (9)	0.0480 (3)	
O5	0.58619 (14)	0.69576 (16)	0.51820 (8)	0.0707 (4)	
O2	0.02213 (12)	0.59831 (16)	0.16524 (10)	0.0721 (4)	
C8	0.22221 (16)	0.51751 (18)	0.28522 (12)	0.0475 (4)	
H8B	0.2773	0.5183	0.3573	0.057*	
H8A	0.1892	0.4184	0.2671	0.057*	
C1	0.52149 (15)	0.59073 (16)	0.36427 (10)	0.0406 (4)	
C4	0.60599 (16)	0.55202 (18)	0.20530 (12)	0.0487 (4)	
H4	0.6219	0.5029	0.1502	0.058*	
C6	0.36474 (16)	0.5264 (2)	0.09100 (11)	0.0533 (4)	
H6A	0.3731	0.4436	0.0511	0.064*	
H6B	0.3589	0.6166	0.0531	0.064*	
C2	0.66126 (16)	0.5530 (2)	0.39249 (12)	0.0550 (4)	
H2A	0.6892	0.4865	0.4503	0.066*	
H2B	0.7126	0.6420	0.4133	0.066*	
O3	0.05036 (15)	0.58840 (17)	0.33329 (12)	0.0822 (5)	
C9	0.10981 (17)	0.6218 (2)	0.26575 (14)	0.0533 (4)	
H9	0.1401	0.7240	0.2748	0.064*	
C7	0.25036 (17)	0.5085 (2)	0.11957 (12)	0.0583 (5)	
H7B	0.1784	0.5690	0.0767	0.070*	
H7A	0.2226	0.4066	0.1133	0.070*	
C3	0.68676 (18)	0.4813 (2)	0.30524 (14)	0.0593 (5)	
H3B	0.7777	0.4911	0.3176	0.071*	
H3A	0.6665	0.3772	0.3024	0.071*	
C11	−0.0810 (2)	0.7013 (3)	0.12986 (19)	0.0959 (8)	
H11A	−0.1268	0.6905	0.0575	0.144*	
H11C	−0.0469	0.7992	0.1449	0.144*	
H11B	−0.1391	0.6837	0.1632	0.144*	
C10	0.0604 (3)	0.6935 (3)	0.40618 (19)	0.1038 (9)	
H10C	0.0242	0.7846	0.3732	0.156*	
H10B	0.1499	0.7079	0.4495	0.156*	
H10A	0.0140	0.6611	0.4464	0.156*	

### Atomic displacement parameters (Å^2^)

**Table:**

	*U*^11^	*U*^22^	*U*^33^	*U*^12^	*U*^13^	*U*^23^
N1	0.0463 (7)	0.0455 (7)	0.0367 (7)	−0.0014 (6)	0.0174 (5)	0.0008 (5)
O1	0.0799 (9)	0.0637 (8)	0.0487 (7)	−0.0237 (7)	0.0295 (7)	−0.0008 (6)
N2	0.0509 (7)	0.0518 (8)	0.0352 (6)	−0.0067 (6)	0.0216 (6)	−0.0049 (6)
C5	0.0503 (8)	0.0298 (7)	0.0356 (7)	0.0001 (6)	0.0216 (6)	0.0037 (6)
O4	0.0738 (9)	0.0590 (8)	0.0535 (7)	0.0098 (6)	0.0316 (6)	−0.0076 (6)
N3	0.0638 (9)	0.0476 (8)	0.0355 (7)	−0.0002 (7)	0.0224 (6)	0.0012 (6)
O5	0.0843 (9)	0.0892 (10)	0.0313 (6)	−0.0007 (8)	0.0149 (6)	−0.0112 (6)
O2	0.0559 (7)	0.0772 (9)	0.0729 (9)	0.0166 (6)	0.0144 (6)	−0.0202 (7)
C8	0.0528 (9)	0.0441 (9)	0.0526 (9)	−0.0010 (7)	0.0284 (7)	0.0035 (7)
C1	0.0515 (9)	0.0403 (8)	0.0330 (7)	0.0005 (6)	0.0200 (6)	0.0033 (6)
C4	0.0579 (10)	0.0517 (10)	0.0464 (9)	−0.0060 (8)	0.0312 (8)	−0.0083 (7)
C6	0.0636 (11)	0.0617 (11)	0.0350 (8)	−0.0065 (8)	0.0197 (7)	−0.0068 (7)
C2	0.0541 (10)	0.0666 (11)	0.0409 (8)	0.0061 (8)	0.0150 (7)	0.0070 (8)
O3	0.0895 (10)	0.0801 (10)	0.1065 (12)	−0.0016 (8)	0.0702 (10)	−0.0059 (9)
C9	0.0553 (10)	0.0478 (10)	0.0646 (11)	−0.0009 (8)	0.0319 (8)	−0.0056 (8)
C7	0.0587 (10)	0.0735 (12)	0.0402 (8)	−0.0090 (9)	0.0166 (8)	−0.0065 (8)
C3	0.0579 (10)	0.0598 (11)	0.0643 (11)	0.0100 (8)	0.0284 (9)	0.0031 (9)
C11	0.0779 (15)	0.114 (2)	0.0836 (16)	0.0394 (14)	0.0185 (12)	−0.0119 (14)
C10	0.132 (2)	0.120 (2)	0.0766 (16)	0.0389 (18)	0.0594 (16)	−0.0029 (14)

### Geometric parameters (Å, °)

**Table:**

N1—C5	1.3488 (19)	C6—H6A	0.9700
N1—C8	1.4579 (19)	C6—H6B	0.9700
N1—C7	1.4777 (19)	C6—C7	1.502 (2)
O1—H1	0.8200	C2—H2A	0.9700
O1—C4	1.400 (2)	C2—H2B	0.9700
N2—C5	1.3412 (19)	C2—C3	1.528 (2)
N2—C4	1.453 (2)	O3—C9	1.404 (2)
N2—C6	1.4566 (19)	O3—C10	1.384 (3)
C5—C1	1.419 (2)	C9—H9	0.9800
O4—N3	1.2643 (18)	C7—H7B	0.9700
N3—O5	1.2751 (17)	C7—H7A	0.9700
N3—C1	1.350 (2)	C3—H3B	0.9700
O2—C9	1.400 (2)	C3—H3A	0.9700
O2—C11	1.419 (2)	C11—H11A	0.9600
C8—H8B	0.9700	C11—H11C	0.9600
C8—H8A	0.9700	C11—H11B	0.9600
C8—C9	1.515 (2)	C10—H10C	0.9600
C1—C2	1.500 (2)	C10—H10B	0.9600
C4—H4	0.9800	C10—H10A	0.9600
C4—C3	1.501 (2)		
			
N1—C5—C1	130.90 (13)	C1—C2—H2A	109.0
N1—C8—H8B	109.2	C1—C2—H2B	109.0
N1—C8—H8A	109.2	C1—C2—C3	112.98 (14)
N1—C8—C9	112.03 (13)	C4—O1—H1	109.5
N1—C7—C6	104.03 (13)	C4—N2—C6	123.77 (12)
N1—C7—H7B	111.0	C4—C3—C2	110.73 (14)
N1—C7—H7A	111.0	C4—C3—H3B	109.5
O1—C4—N2	111.53 (14)	C4—C3—H3A	109.5
O1—C4—H4	109.5	C6—C7—H7B	111.0
O1—C4—C3	109.90 (14)	C6—C7—H7A	111.0
N2—C5—N1	110.37 (13)	H6A—C6—H6B	109.3
N2—C5—C1	118.73 (14)	C2—C3—H3B	109.5
N2—C4—H4	109.5	C2—C3—H3A	109.5
N2—C4—C3	106.79 (13)	H2A—C2—H2B	107.8
N2—C6—H6A	111.4	O3—C9—C8	109.02 (15)
N2—C6—H6B	111.4	O3—C9—H9	110.4
N2—C6—C7	101.74 (12)	O3—C10—H10C	109.5
C5—N1—C8	125.00 (12)	O3—C10—H10B	109.5
C5—N1—C7	108.64 (13)	O3—C10—H10A	109.5
C5—N2—C4	122.00 (13)	C9—O2—C11	114.29 (15)
C5—N2—C6	111.22 (13)	C9—C8—H8B	109.2
C5—C1—C2	120.14 (13)	C9—C8—H8A	109.2
O4—N3—O5	119.82 (13)	C7—C6—H6A	111.4
O4—N3—C1	123.22 (13)	C7—C6—H6B	111.4
N3—C1—C5	122.62 (14)	H7B—C7—H7A	109.0
N3—C1—C2	116.43 (14)	C3—C4—H4	109.5
O5—N3—C1	116.87 (14)	C3—C2—H2A	109.0
O2—C9—C8	107.03 (13)	C3—C2—H2B	109.0
O2—C9—O3	109.65 (15)	H3B—C3—H3A	108.1
O2—C9—H9	110.4	H11A—C11—H11C	109.5
O2—C11—H11A	109.5	H11A—C11—H11B	109.5
O2—C11—H11C	109.5	H11C—C11—H11B	109.5
O2—C11—H11B	109.5	C10—O3—C9	116.47 (18)
C8—N1—C7	117.14 (13)	H10C—C10—H10B	109.5
C8—C9—H9	110.4	H10C—C10—H10A	109.5
H8B—C8—H8A	107.9	H10B—C10—H10A	109.5
			
N1—C5—C1—N3	−25.8 (2)	O5—N3—C1—C2	−7.5 (2)
N1—C5—C1—C2	164.90 (15)	C8—N1—C5—N2	150.28 (14)
N1—C8—C9—O2	68.81 (18)	C8—N1—C5—C1	−29.3 (2)
N1—C8—C9—O3	−172.66 (14)	C8—N1—C7—C6	−164.17 (14)
O1—C4—C3—C2	61.14 (19)	C1—C2—C3—C4	38.9 (2)
N2—C5—C1—N3	154.74 (15)	C4—N2—C5—N1	169.82 (14)
N2—C5—C1—C2	−14.6 (2)	C4—N2—C5—C1	−10.6 (2)
N2—C4—C3—C2	−59.99 (19)	C4—N2—C6—C7	−178.50 (15)
N2—C6—C7—N1	19.23 (18)	C6—N2—C5—N1	8.77 (17)
C5—N1—C8—C9	135.02 (15)	C6—N2—C5—C1	−171.63 (13)
C5—N1—C7—C6	−15.63 (19)	C6—N2—C4—O1	86.66 (18)
C5—N2—C4—O1	−71.99 (18)	C6—N2—C4—C3	−153.26 (15)
C5—N2—C4—C3	48.10 (19)	C7—N1—C5—N2	4.82 (17)
C5—N2—C6—C7	−17.84 (18)	C7—N1—C5—C1	−174.72 (15)
C5—C1—C2—C3	−1.2 (2)	C7—N1—C8—C9	−82.11 (18)
O4—N3—C1—C5	−0.8 (2)	C11—O2—C9—C8	−171.90 (18)
O4—N3—C1—C2	168.94 (14)	C11—O2—C9—O3	70.0 (2)
N3—C1—C2—C3	−171.20 (15)	C10—O3—C9—O2	−129.9 (2)
O5—N3—C1—C5	−177.19 (14)	C10—O3—C9—C8	113.2 (2)

### Hydrogen-bond geometry (Å, °)

**Table:**

*D*—H···*A*	*D*—H	H···*A*	*D*···*A*	*D*—H···*A*
O1—H1···O5^i^	0.82	1.89	2.6966 (16)	169.

Symmetry codes: (i) *x*, −*y*+3/2, *z*−1/2.
